# Small but Smart: On the Diverse Role of Small Proteins in the Regulation of Cyanobacterial Metabolism

**DOI:** 10.3390/life10120322

**Published:** 2020-12-01

**Authors:** Fabian Brandenburg, Stephan Klähn

**Affiliations:** Helmholtz Centre for Environmental Research—UFZ, 04318 Leipzig, Germany; fabian.brandenburg@ufz.de

**Keywords:** small proteins, metabolic regulation, biotechnology

## Abstract

Over the past few decades, bioengineered cyanobacteria have become a major focus of research for the production of energy carriers and high value chemical compounds. Besides improvements in cultivation routines and reactor technology, the integral understanding of the regulation of metabolic fluxes is the key to designing production strains that are able to compete with established industrial processes. In cyanobacteria, many enzymes and metabolic pathways are regulated differently compared to other bacteria. For instance, while glutamine synthetase in proteobacteria is mainly regulated by covalent enzyme modifications, the same enzyme in cyanobacteria is controlled by the interaction with unique small proteins. Other prominent examples, such as the small protein CP12 which controls the Calvin–Benson cycle, indicate that the regulation of enzymes and/or pathways via the attachment of small proteins might be a widespread mechanism in cyanobacteria. Accordingly, this review highlights the diverse role of small proteins in the control of cyanobacterial metabolism, focusing on well-studied examples as well as those most recently described. Moreover, it will discuss their potential to implement metabolic engineering strategies in order to make cyanobacteria more definable for biotechnological applications.

## 1. Introduction

In nature, proteins are one of the most versatile classes of biological compounds. They serve multiple purposes as structural components, enzymes, membrane transporters, signaling molecules or regulatory factors. Given their tremendous variability in fulfilling tasks in all aspects of life, it is not surprising that proteins come in a manifold of sizes and shapes. For example, they can be single domain proteins or a part of huge protein complexes. The biggest so-far known example that is not part of a multiunit structure is the protein Titin, which is part of vertebrate muscles [1]. Depending on the splice variant, Titin has a size of 27,000–35,000 amino acids and contains over 300 domains [2]. On the contrary, the protein Tal, which was found in *Drosophila melanogaster* is composed of only 11 amino acids [3]. Albeit being so small, it is involved in controlling gene expression and tissue folding and hence, is the shortest functional protein described so far.

It is known that the mean protein length of bacteria is 40–60% shorter than of eukaryotes [4]. Moreover, it was found that up to 16% of all proteins in a prokaryotic organisms might be actually smaller than 100 amino acids [5]. Consequently, more and more studies are suggesting that likely hundreds of small proteins are synthesized in bacterial cells and serve important structural and regulatory functions [6]. Of course, some of these small proteins are known for decades and well-studied, such as, for example, thioredoxins, which play important roles as antioxidants in almost all organisms, not only prokaryotes [7,8,9]. However, genes encoding small proteins are likely to be overlooked even in modern genome annotations because the minimal cutoff for small open reading frames is typically set to 100 amino acids [10,11]. In turn, this indicates the existence of a whole, unexplored universe of small proteins to be discovered in bacteria, which is especially exemplified by the phylum of cyanobacteria.

Cyanobacteria are the only prokaryotes performing oxygenic photosynthesis. To conduct and maintain their complex photosynthetic machinery, which is composed of several functionally related protein complexes, cyanobacteria use a plethora of small proteins. Some examples like Psb27 have been shown to be important in photosystem II (PSII) repair [12], while others like PetP are involved in stress adaptation of the photosynthetic electron transport chain [13]. In fact, more than 10 proteins smaller than 50 amino acids have been characterized to be important for the function of PSII alone [14,15]. Additionally, 293 candidate-genes for proteins smaller than 80 amino acids have been identified in the cyanobacterial model organism *Synechocystis* sp. PCC 6803 (hereafter *Synechocystis*), indicating that cyanobacteria provide a paradigm for the utilization of small proteins and hence, the functional characterization of bacterial micro-proteomes [16].

This review highlights further prominent examples of small proteins in cyanobacteria beyond the photosynthetic apparatus, i.e., those exercising a regulatory function related to primary metabolism. However, in the literature, different definitions for the term ‘small protein’ exist. Some studies limit the term to proteins ≤85 amino acids [17], while others also include proteins up to a size of 200 amino acids [18]. Some authors also use the term ‘microproteins’ which is typically defined as proteins up to a size of 80 amino acids [16]. In this review, we did not set a specific cut-off for the size of considered proteins, but focused on those candidates that regulate metabolic pathways via protein–protein interaction, among which various truly small proteins of only a few kDa are found. Finally, the potential of small proteins as an add-on for the currently existing molecular toolbox for metabolic engineering in cyanobacteria is discussed.

## 2. Light Regulation of the Calvin-Benson Cycle by the Small Protein CP12

In cyanobacteria, the Calvin–Benson cycle (CB) is the central pathway for the generation of biomass as it fixes CO_2_ by using energy (ATP) and reduction equivalents (NADPH) derived from the photosynthetic electron transport chain. In darkness however, cyanobacteria need to oxidize carbohydrates to cover their needs of ATP and reductive power. For a long time, it was believed that the Embden–Meyerhof–Parnas pathway (glycolysis) and the oxidative pentose phosphate pathway (OPP) are the only pathways for carbohydrate oxidation in cyanobacteria. Only quite recently it could be shown that the Entner–Doudoroff (ED) pathway is also active [19]. However, switching from a phototrophic to a heterotrophic mode cannot be achieved by simply activating the respective pathways. Both glycolysis and the OPP share several intermediates with the CB and thus may form a futile cycle when operated at the same time [19]. In order to prevent this, these pathways as well as the CB respond to several signals that trigger a regulation of their activities. For the CB, these signals are thioredoxin, pH, the levels of magnesium and certain metabolites such as fructose 6-phosphate and sedoheptulose 7-phosphate [20]. Some of these signals are transmitted into the regulation of enzyme activity by protein-protein interactions. In plants, the key enzyme of the CB, ribulose-1,5-bisphosphate-carboxylase/-oxygenase (RuBisCO) is directly regulated by an enzyme called RuBisCO activase with a chaperone-like function [21]. However, this does not appear to be a general mechanism among cyanobacteria, as the respective enzyme could be found only in a few species [21].

**Figure 1:**
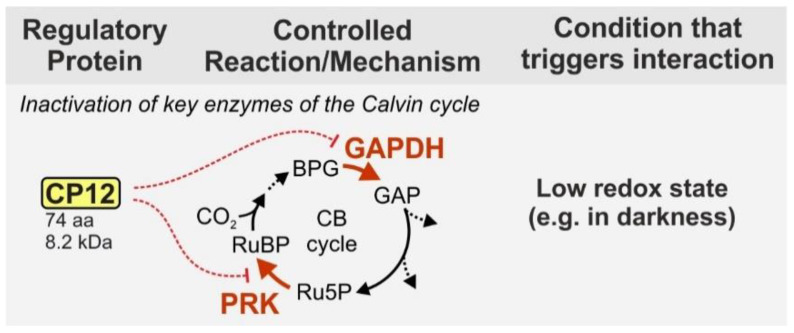
Function of the small protein CP12 in cyanobacteria. CP12 inhibits the CB cycle via complex formation with two key enzymes, PRK and GAPDH. The complex formation is initiated by a low redox status of the cell (e.g., low availability of reduction equivalents) and dinucleotide availability signals (e.g., changes in the NADP(H)/NAD(H) ratio) like they occur during transition from light to darkness. The protein size is given for the model strain *Synechocystis*. BPG—1,3-bisphopshoglycerate, GAP—glyceraldehyde 3-phosphate, Ru5P—ribose 5-phosphate, RuBP—ribulose 1,5 bisphosphate, GAPDH—glyceraldehyde 3-phosphate dehydrogenase, PRK—phosporibulokinase, CB cycle—Calvin–Benson cycle.

On the contrary, the small ‘chloroplast protein of 12 kDa’ (CP12—74 aa, 8.3 kDA in *Synechocystis*) seems to be universally distributed among organisms performing oxygenic photosynthesis. CP12 presents an additional layer of regulation [22]. Under dark conditions, the protein forms a supramolecular complex with the enzymes glyceraldehyde-3-phosphate dehydrogenase (GAPDH) and phosphoribulokinase (PRK) and thereby inhibits their activity [23,24] (Figure 1). Both enzymes are important regulatory points of the CB because they act at the branching points of the CB and OPP. In an ATP-consuming step PRK produces the RuBisCO substrate ribulose 1,5-bisphosphate from the OPP intermediate ribulose 5-phosphate. GAPDH uses NADPH to produce glyceraldehyde 3-phosphate, which is the main exit point of the CB and also part of the OPP. Inhibition of these two enzymes therefore helps the cell to preserve energy, when the photosynthetic light reaction is not active.

CP12 contains four conserved cysteine residues, two at each end. Under oxidizing conditions these cysteine residues form two terminal loops, which enable complex formation with first GADPH and then PRK [25,26,27]. Binding of GAPDH leads to a conformational change of CP12, which results in an extensive negative charge potential on its molecular surface. This negative charge potential mediates the binding of its N-terminal loop with PRK [25]. The formed complex drastically reduces the activity of both enzymes [28]. The complex formation is modulated by the ratio of NADP(H) to NAD(H), which decreases to almost 50% upon transition from light to darkness, and the redox status of the cell [27,29]. Compared to wild-type (WT), CP12-deficient cells are unable to regulate the CB and hence, grow slower under fluctuating light conditions, while there is no difference under continuous light conditions [29]. Interestingly, cyanophages infecting marine picocyanobacteria of the genera *Prochlorococcus* and *Synechococcus* have been shown to express functional CP12 in their host cells, likely to shut down the CB and use the host’s production of NADPH to fuel their own deoxynucleotide biosynthesis for replication [30].

## 3. Control of Glutamine Synthetase by Proteinaceous Inactivating Factors Unique to Cyanobacteria

Besides light and CO_2_, nitrogen is another important environmental factor determining cyanobacterial growth. While some cyanobacteria are able to fix dinitrogen gas [31,32] most strains rely on the uptake of reduced nitrogen sources from their environment. Cyanobacteria can utilize a variety of nitrogen sources such as nitrate, nitrite, ammonium, urea, cyanate and some amino acids (such as arginine, glutamine and glutamate) [33,34,35,36,37]. Nevertheless, ammonium is preferred due to a lower energy demand for its assimilation compared to other nitrogen sources [35,38]. The manifold of nitrogen sources as well as their changing availability requires a well-orchestrated regulatory network of nitrogen metabolism in cyanobacteria.

**Figure 2:**
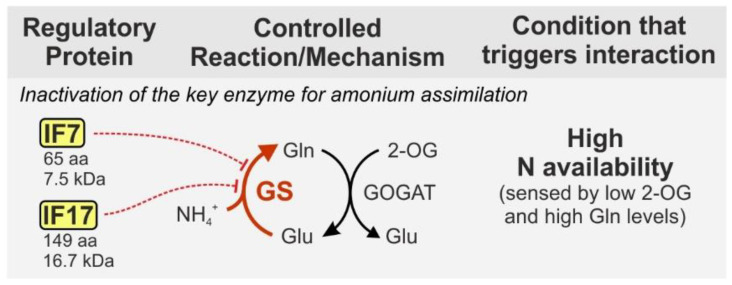
Inactivation of cyanobacterial GS by the interaction with small proteins. Most cyanobacteria harbor two homologous inactivating factors: IF7 and IF17, encoded by the genes *gifA* and *gifB*. The interaction with GS does not require a metabolic signal, hence IF7 and IF17 synthesis is tightly regulated at the transcriptional level by NtcA, which is further assured by regulatory RNAs acting at the post-transcriptional level. IF synthesis is stimulated by low 2-OG levels as well as high Gln levels (see text). Protein sizes are given for *Synechocystis* in which the proteins have initially been discovered. GS—glutamine synthetase, GOGAT—glutamine oxoglutarate aminotransferase (glutamate synthase), Gln—glutamine, Glu—glutamate, 2-OG—2-oxoglutarate.

Assimilated nitrate and nitrite are reduced inside the cell and the resulting ammonium is incorporated into carbon skeletons via glutamate dehydrogenase and the glutamine synthetase/glutamate synthase cycle (GS/GOGAT) [35]. The key enzyme GS is well known to be regulated by reversible adenylylation in several bacterial species [39]. By contrast, the cyanobacterial GS is regulated by interaction with small proteins, the so-called GS inactivating factors (IFs) two of which have been identified in *Synechocystis*: IF7 (65 aa, 7.5 kDa) and IF17 (149 aa, 16.7 kDa) [40]. Both IFs are synthesized under nitrogen-rich conditions and specifically bind to GS causing complete enzyme inactivation (Figure 2).

GS activity is exclusively regulated by the abundance of IF7 and IF17 in the cell [40]. Consequently, their synthesis is target of tight control mechanisms, at the transcriptional and post-transcriptional level. For instance, the corresponding genes, *gifA* and *gifB* are repressed by NtcA [41], a universal transcriptional regulator of nitrogen assimilation in cyanobacteria, which is active and binds DNA under low-nitrogen conditions [42]. Accordingly, *gifA* and *gifB* expression rapidly increases in response to ammonium upshifts. Moreover, IF7 synthesis is negatively regulated by the small RNA NsiR4, which interacts with the *gifA* mRNA and interferes with its translation [43]. In addition, another unique RNA-dependent mechanism has evolved, namely a glutamine riboswitch, which is present in the 5’UTR of the *gifB* transcript. It tightly controls IF17 synthesis in response to a glutamine threshold that is passed when GS activity, i.e., glutamine synthesis exceeds a certain level [44]. The peculiarities of the complex regulation of GS by direct interaction with the small proteins IF7 and IF17 are also reviewed elsewhere in more detail [45]. However, it should be noted that although IFs are unique to cyanobacteria, GS regulation by small proteins is not restricted to this group *per se*. For instance, GS activity is stimulated by complex formation with the 23 aa peptide sP26 and further modulated in a 2-oxoglutarate (2-OG) dependent manner by the 114 aa protein GlnK_1_ in the archaeal model *Methanosarcina mazei* [46,47]. Both proteins are not related to the cyanobacterial IFs, which greatly exemplifies how widespread and versatile regulatory mechanisms by small proteins are even when targeting the same enzyme.

## 4. Control of the Key Enzyme of Arginine Synthesis by Direct Interaction with the PII Protein

**Figure 3:**
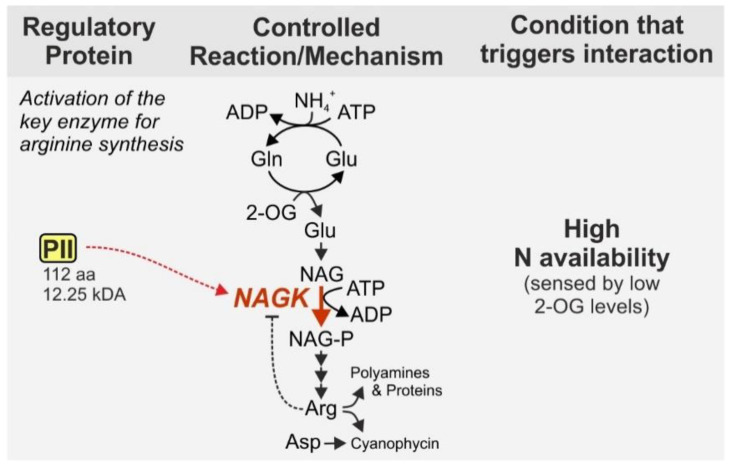
Regulation of N-acetyl-*L*-glutamate kinase (NAGK) by complex formation with the PII protein. NAGK is target of feedback inhibition by arginine, which is however minimized when PII interacts with NAGK. Thereby PII interaction enhances the flux through the rate-limiting step of arginine synthesis, which also impacts synthesis of the N storage compound cyanophycin. We chose NAGK as a prime example for the regulation of enzymes by direct interaction with the PII protein. Nevertheless, it should be noted that PII interacts with various other proteins including enzymes [56]. NAG—N-acetyl-L-glutamate, NAG-P—NAG phosphate, Arg—arginine, Asp—aspartate, Gln—glutamine, Glu—glutamate, 2-OG—2-oxoglutarate.

The PII signaling protein, a homolog to the aforementioned GlnK_1_, fulfills important regulatory functions and hence, is widely distributed, i.e., present in archaea, bacteria and chloroplasts of plants [48]. With a size of 112 aa and 12.25 kDa in *Synechocystis* it can be defined as a small protein and will be highlighted even though it is also present in other bacteria. In cyanobacteria, the PII protein namely has distinctive regulatory functions. Unlike other bacterial phyla, cyanobacteria possess only one copy of PII [49]. Among other functions, the cyanobacterial PII protein regulates arginine synthesis by binding to the *N*-acetyl-*L*-glutamate kinase (NAGK), which catalyzes the second, rate limiting step of arginine synthesis from glutamate [50]. In addition to its incorporation into proteins, arginine is important as precursor for the nitrogen storage compound cyanophycin, which is a copolymer of aspartate and arginine [42,51]. NAGK is subject to strong feedback inhibition by arginine [52,53,54]. However, complex formation with PII prevents feedback inhibition of NAGK by arginine and thus strongly enhances the activity of the enzyme [55] (Figure 3). 

The activity of PII itself is regulated by phosphorylation, which is in contrast to heterotrophic bacteria, where PII proteins are controlled by uridylylation [38,57]. The phosphorylation status of PII correlates with the nitrogen status of the cell, i.e., fully phosphorylated PII protein is present in cells grown under nitrogen depleted conditions [38,58]. In contrast, the PII protein is completely dephosphorylated in cells grown in the presence of their preferred nitrogen source (ammonium) but gets phosphorylated when cells are using less preferred nitrogen sources like nitrate or nitrite [59]. The phosphorylation status of PII is dependent on the binding of both ATP and 2-OG which lead to a conformational change of PII that is recognized by the modifying enzymes PII-P phosphatase and PII kinase [60,61,62]. Here, 2-OG serves as a proxy for the nitrogen status of the cell, because due to the activity of the GS/GOGAT cycle the level of 2-OG correlates well with the nitrogen amount that is externally available for the cell [56,60]. Its function as a key regulatory protein is underlined by the fact that recombinant strains of *Synechococcus elongatus* PCC 7942 lacking functional PII are unable to adapt to changing environmental conditions, in particular to changes in nitrogen availability and changes from ammonium to other nitrogen sources, but also to changes in CO_2_ concentrations and light intensities [58]. 

Furthermore, unphosphorylated PII is hypothesized to regulate the uptake of nitrate and nitrite at the post-translational level [49,63]. Likely the PII protein binds directly to the transport protein and thereby inhibits transport [64,65]. In fact, it has been shown that PII is involved in the uptake of ammonium by interaction with the ammonium permease Amt1, inhibits the uptake of nitrate by interaction with the NrtC and NrtD subunits of the nitrate/nitrite transporter NrtABCD, and interacts with the UrtE subunit of the urea transporter UrtABCDE [66]. Additionally, the uptake of bicarbonate is altered in PII-knockout mutants of *Synechococcus elongatus* PCC 7942 [67] and no uptake of methylammonium could be measured in PII-deficient cells of *Synechococcus elongatus* PCC 7942 [58].

In addition, multiple other interaction partners of PII have been identified like the enzyme acetyl-CoA carboxylase (ACCase) [68] or the membrane protein PamA [69]. Some of these interaction partners are small proteins themselves, which in turn exercise metabolic control (see below). 

## 5. PII as an Antagonist for the Interaction of Small Proteins with Key Factors that Control Metabolic Fluxes 

**Figure 4:**
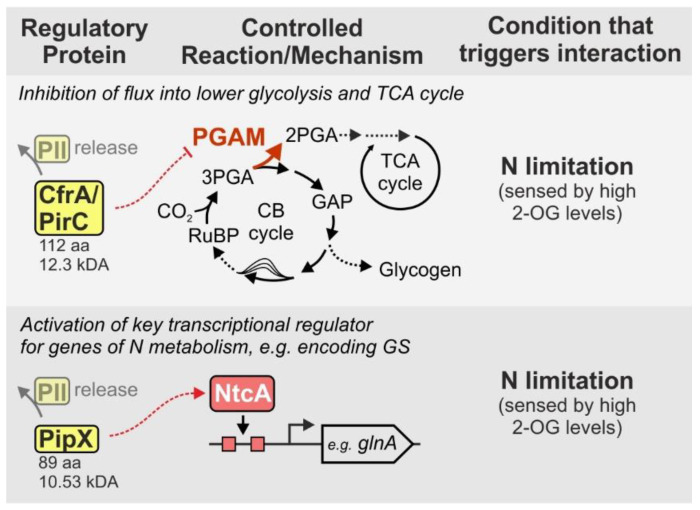
PII modulates and controls the interaction of further small proteins with key elements of cyanobacterial metabolism. Under conditions of sufficient nitrogen both, PipX and CfrA/PirC, form a complex with PII. Under nitrogen limitation, PII releases the small proteins that in turn interact with other target proteins thereby controlling their activity, e.g., enhancing DNA binding affinity of NtcA or inhibiting 3-phosphoglycerate mutase (PGAM).

Most recently, two independent studies identified another interesting example for the regulation of metabolic processes by a small protein in cyanobacteria [70,71]. Both studies suggested different names for the same protein, namely carbon flow regulator A (CfrA, Muro-Pastor et al., 2020) and PII-interacting regulator of carbon metabolism (PirC, Orthwein et al. 2020), hence we refer to both names in this case. The protein is highly conserved and can be found in almost all cyanobacterial species [70,71]. Free CfrA/PirC (112 aa, 12.27 kDa in *Synechocystis*) binds and inhibits 3-phosphoglycerate mutase (PGAM), an enzyme whose activity is directing carbon flux from the CB cycle towards lower glycolysis [71] (Figure 4). Hence, recombinant strains showing overproduction of CfrA/PirC accumulated excessive amounts of glycogen, while respective knockout mutants were unable to accumulate glycogen even under nitrogen depletion [70]. Instead, those strains accumulated polyhydroxybutyrate (PHB), another carbon storage compound of cyanobacteria, which derives from acetyl-CoA, i.e., reactions downstream of the one that is catalyzed by PGAM [71]. Remarkably, both studies reported complex formation of CfrA/PirC with the PII protein, which is tuned by the 2-OG level [70,71]. Thereby, PII and its interaction with CfrA/PirC determines whether or not PGAM is inhibited by interacting with the small protein. Like other bacterial groups, cyanobacteria sense the nitrogen status via the levels of 2-OG and one of the main routes of newly fixed CO_2_ is the synthesis of 2-OG for the assimilation of nitrogen via the GS-GOGAT cycle [59,72]. In the presence of low 2-OG levels, which signals a sufficient nitrogen status, CfrA/PirC interacts with PII. The PII-CfrA/PirC complex disassembles in presence of high 2-OG levels (e.g., under nitrogen limitation) and the released small protein then inhibits PGAM via protein-protein interaction. 

A similar interplay between PII and a small protein refers to the activity of the transcription factor NtcA that regulates genes majorly encoding elements of nitrogen metabolism in cyanobacteria (for a review see [42]). The small protein PipX (89 aa, 10.53 kDa in *Synechocystis*) interacts with either NtcA or PII depending on the nitrogen status of the cell, sensed via 2-OG [73]. Under nitrogen sufficient conditions, i.e., low 2-OG levels, PipX is bound to PII and NtcA is inactive. In contrast, under nitrogen limiting conditions, PipX is released and interacts with NtcA. Thereby it functions as a coactivator required to enhance 2-OG-dependent binding of NtcA to its recognition sequence [73]. Albeit not exclusively, NtcA commonly acts as transcriptional activator [74] and hence, DNA binding that is triggered by 2-OG and PipX, induces sufficient expression of nitrogen assimilatory genes upon nitrogen limitation, e.g., the *glnA* gene encoding GS (Figure 4).

The structural basis for different binding preferences of PipX has also been investigated [75,76]. However, just like PII, PipX might have even more targets and functions than currently known. For instance, binding of PipX to PII facilitates the extension of the C-terminal region of PipX and thus allow for different interaction partners than the unbound PipX protein [77]. Moreover, it was found that under nitrogen-starving conditions, only 25% of the present PipX in *Synechococcus elongatus* PCC 7942 are needed to bind 100% of the present NtcA leaving 75% of the PipX protein unbound or potentially bound to unknown targets [77]. For instance, it has been shown that under nitrogen-sufficient conditions PII-bound PipX interacts with the GntR-like regulator PlmA [78]. Nevertheless, for the distinctive features of the cyanobacterial PipX protein we refer to a recent review [79].

In addition to the control that PII executes via direct interaction with enzymes such as NAGK, it appears as an antagonist of further small proteins that fulfill crucial regulatory functions in cyanobacterial metabolism. The two given examples are maybe only the tip of the iceberg and hence, further small proteins could function in a similar way. This is supported by the fact that another small protein, encoded by the *ssr0692* gene in *Synechocystis* has been copurified with PII previously [66]. Very recently, it was shown that Ssr0692 (51 aa, 5.8 kDa) is required to balance the synthesis of arginine and several other key amino acids under fluctuating N conditions. Indeed, the protein was confirmed to interact with PII and was hence named PII-interacting regulator of arginine synthesis (PirA, [80]).

## 6. Further Examples of Small Protein Regulators Affecting the Activity of Enzymes or Transporters

**Figure 5:**
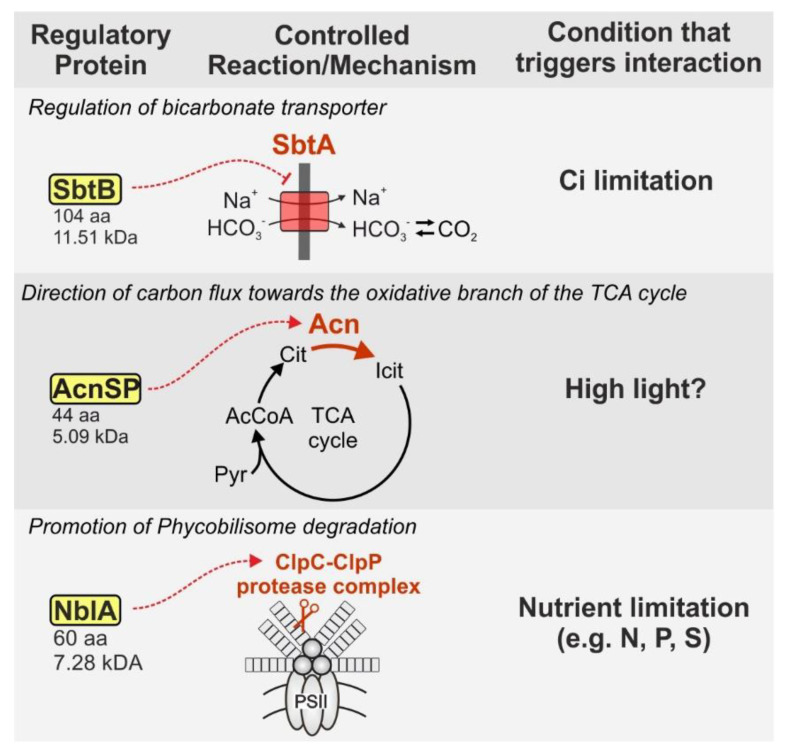
Further examples of small protein regulators affecting the activity of enzymes or transporters by direct interaction. The given protein sizes refer to *Synechocystis*. AcCoA—acetyl-CoA, Acn—aconitase, Ci—inorganic carbon, Cit—citrate, Icit—Isocitrate, Pyr—pyruvate, PSII—photosystem II, TCA cycle—tricarboxylic acid cycle.

In addition to the PII protein, which is highly conserved in structure and function between different species, there are multiple so-called PII-like proteins that lack multiple consensus sequences and show rather low sequence identity [81]. One example is the protein GlnK, which is also involved in the control of nitrogen assimilation and GS regulation in various prokaryotes [47,82,83]. In cyanobacteria, the PII-like protein SbtB (104 aa, 11.51 kDa in *Synechocystis*) has been shown to interact with the bicarbonate transporter SbtA, which thereby likely has an inhibitory effect [84,85] (Figure 5). Structural analysis of SbtB revealed that increasing concentrations of ATP are one signal initiating the release of SbtB from SbtA [86]. This example shows that in addition to the complex regulatory network that has been unraveled for PII over the past decades, there might be a plethora of PII-like proteins, which could also serve important regulatory functions. The question remains if these proteins have a similar manifold of interaction partners, and with this, an equivalent regulatory importance that has been shown for PII.

Another very recent example for a small protein regulator affecting enzyme activity is AcnSP (44 aa, 5.09 kDa in *Synechocystis*), which is also the smallest protein presented in this review. AcnSP was proposed to stimulate the activity of its binding partner aconitase (Acn, Figure 5), and thereby impacts carbon flux towards the oxidative part of the tricarboxylic acid (TCA) cycle [87]. While the structural mechanism of protein–protein interaction and the exact biological function of AcnSP require further investigation, a potential link to high light adaptation has been already revealed [87].

Upon nutrient limitation, a common reaction is the degradation of the phycobilisomes, the major light-harvesting complexes of cyanobacteria. Several small proteins are involved in the process. Knockout of the corresponding genes leads to a nonbleaching (nbl) phenotype upon nitrogen starvation. The small protein NblA (60 aa, 7.28 kDa) was the first discovered protein involved in the process. The transcription of *nblA* increases moderately in phosphorous-deprived cells, but shows a much stronger response in sulfur- or nitrogen-deprived cells, leading to a partial or complete degradation of phycobilisomes, respectively [88]. Analysis of the crystal structure of NblA revealed that the protein is present as a homodimer and interacts via the C-terminus with the phycobilisomes [89]. Further studies revealed that in addition to the phycobilisome, NblA binds ClpC at the same time [90]. ClpC is part of the ClpC•ClpP protease complex. The interaction of NblA with ClpC initiates protein degradation by guiding the protease machinery to the phycobilisome [90] (Figure 5). Another small protein named NblB (233 aa, 25.51 kDa) functions analog to NblA and knockouts of *nblB* are also unable to to degrade phycobilisomes under nutrient limiting conditions [91]. In contrast to NblA, NblB is expressed similarly in both cells facing nutrient limitation and nutrient-sufficient conditions [91], indicating another, yet unknown, layer of regulation for this protein. Interestingly, recent results show that NblA and NblB not only facilitate degradation of phycobilisomes, but also binding and rearrangement of chromophores to phycobilisomes [92]. Thus, NblA and NblB might play an important role for short-term adjustments of the photocomplexes to optimize light harvesting under changing light conditions in natural environments.

Most recently, an additional small protein termed NblD (66 aa, 7.09 kDa) was described [93]. The gene locus for NblD was first discovered in a transcriptome study as a highly transcribed but not annotated region and determined as transcriptional unit (TU) 728 [94]. Later, it was discovered that TU728 accumulates upon nitrogen limitation and encodes a small protein, which was named NsiR6 [16]. Studies with knockout strains revealed a role in phycobilisome degradation similar to NblA and the β-subunit of phycocyanin as interaction target. Accordingly, it was named NblD. However, the exact function of NblD and the interplay with the other Nbl-proteins remains to be unraveled at this point [93].

## 7. The Potential of Small Proteins for Metabolic Engineering and Biotechnological Applications 

Interest in cyanobacteria as host organisms for biotechnological applications has increased steadily over the past decade [95,96,97,98,99]. To date, more than 20 chemicals have been synthesized in cyanobacteria directly from CO_2_, e.g., 1,2-propanediol, cyclohexanol, ethanol, isobutyraldehyde, isobutanol, 1-butanol, isoprene, ethylene, hexoses, cellulose, mannitol, lactic acid and fatty acids [96,98,100,101,102]. Theoretically, every chemical that can be produced by heterotrophic bacteria can also be produced photoautotrophically using cyanobacteria. Thereby, a direct production based on photosynthetic CO_2_ fixation is hypothesized to be beneficial over the intermediate CO_2_ fixation into biomass, required as source for a heterotrophic production process [98,103].

The growing interest in the biotechnological utilization of cyanobacteria comes with an increasing demand for advanced molecular tools for the genetic engineering of cyanobacteria, which lack far behind the toolset that is available, e.g., for *E. coli* [104,105]. Besides tools for the predictable control of gene expression like promoters [106], this also includes ways to reroute the endogenous carbon flux towards the desired reaction, e.g., blocking of competing pathways or the synthesis of storage compounds [107]. Typically, this is achieved by knocking out the gene encoding the respective synthesis, for example by insertional inactivation with an antibiotic resistance cassette. One major drawback of knockout mutants is the permanent loss of a specific gene function, which might be disadvantageous under certain conditions, in particular in the long-term under natural, i.e., steadily fluctuating conditions. For instance, knockout mutants of glycogen biosynthesis show impaired growth under fluctuating light conditions [108]. This might be problematic, for example, in outdoor bioreactors during phases of biomass production, in which product formation is not the primary interest. Another approach to control gene regulation in bioengineered strains is RNA silencing by small antisense RNA (asRNA)—a technique that has been established also in cyanobacteria as an alternative method for permanent knockouts [109,110]. In comparison to permanent knockouts, asRNA constructs can be controlled, for example, by an inducible promoter independently or in the same way as the respective genes for the production process. However, RNA silencing approaches can be prone to off-target effects and genetic instability of the asRNA construct in subsequent generations [111]. The latter is especially critical in photoautotrophic bacterial systems with short generation times and ideally long or even continuous cultivation intervals.

As pointed out in this review, small proteins are able to sense and implement different signals, bind several interaction partners at the same time and thereby play important regulatory roles in diverse metabolic pathways. Hence, small proteins might pose an interesting addition to the molecular tool set of cyanobacteria. In fact, a few examples can already be found in literature. For instance, the aforementioned CP12 protein has been used to engineer carbon metabolism of the cyanobacterium *Synechococcus elongatus* PCC 7942, i.e., to improve CO_2_ fixation and, together with other modifications, to increase the production of 2,3-butanediol [112]. Similarly, the carbon flow regulator CfrA/PirC together with two other modifications has been utilized to increase the production yield of the plastic alternative polyhydroxybutyrate (PHB) in *Synechocystis* from 15 to 63% per cell dry weight (CDW) and even to 81% with additional feeding of acetate [113]. In addition, targeted engineering of existing regulatory proteins may allow the generation of variants exhibiting different binding characteristic compared to the native protein. The principle has already been demonstrated for variants of the PII protein. For instance, variants mimicking either the phosphorylated or the unphosphorylated state of the protein resulted in strains with different nitrogen uptake characteristics compared to WT cells [63]. Moreover, other variants led to a constitutive interaction with NAGK, accompanied by enhanced arginine synthesis and cyanophycin accumulation [114]. Altogether, the greater chemical diversity of proteins compared to small pieces of RNA may result in a control mechanism that is less prone to off targets and more stable over several generations.
